# Qianlie Xiaozheng Decoction Induces Autophagy in Human Prostate Cancer Cells via Inhibition of the Akt/mTOR Pathway

**DOI:** 10.3389/fphar.2018.00234

**Published:** 2018-04-04

**Authors:** Yuehua Xu, Xueting Cai, Bin Zong, Rui Feng, Yali Ji, Gang Chen, Zhongxing Li

**Affiliations:** ^1^Zhenjiang Hospital of Chinese Traditional and Western Medicine, Zhenjiang, China; ^2^Affiliated Hospital of Integrated Traditional Chinese and Western Medicine, Nanjing University of Chinese Medicine, Nanjing, China; ^3^Laboratory of Cellular and Molecular Biology, Jiangsu Province Academy of Traditional Chinese Medicine, Nanjing, China; ^4^College of Veterinary Medicine, Yangzhou University, Yangzhou, China

**Keywords:** Qianlie Xiaozheng decoction, autophagy, prostate cancer, Akt/mTOR pathway, PC3 cell

## Abstract

Qianlie Xiaozheng decoction (QLXZD), a traditional Chinese medicinal formula, has been used clinically to treat advanced prostate cancer (PCa) for more than 10 years. However, experimental evidence supporting its efficacy is lacking. Here, we investigated the anticancer properties and molecular mechanism of QLXZD *in vitro* in a human PCa cell line (PC3) and *in vivo* using PC3 xenografts in nude mice. We confirmed the antineoplastic activity of QLXZD by analyzing cell viability and tumor volume growth, which decreased significantly compared to that of the controls. Autophagy following QLXZD treatment was detected morphologically using transmission electron microscopy and was confirmed by measuring the expression of autophagy markers (LC3-II and p62) using fluorescence analysis, flow cytometry, and western blotting. Increasing autophagic flux induced by QLXZD was monitored via pmCherry-GFP-LC3 fluorescence analysis. QLXZD-induced autophagic cell death was alleviated by the autophagy inhibitors, 3-methyl adenine and hydroxychloroquine. We evaluated the total expression and phosphorylation levels of proteins involved in the Akt/mTOR pathway regulating autophagy. Phosphorylation of Akt, mTOR, and p70S6K, but not total protein levels, decreased following treatment. This is the first study to demonstrate the autophagy-related mechanistic pathways utilized during QLXZD-mediated antitumor activity both *in vitro* and *in vivo*. These findings support the clinical use of QLXZD for PCa treatment.

## Introduction

Prostate cancer is one of the most prevalent malignant tumors as well as the second leading cause of cancer-related deaths in men in Western countries, with appropriately 15% of men having PCa during their lifetime (Kar et al., 2016). Androgen deprivation therapy, a treatment for PCa, shows obvious effects at the beginning of treatment, but after 14–30 months, most patients develop HIPC (Thy et al., 2015; Raymond et al., 2017). The median survival for HIPC patients is less than 20 months, and there is currently no effective treatment or cure (Raymond et al., 2017). Therefore, it is essential to elucidate the mechanisms underlying the onset of PCa and to develop novel treatment options for advanced PCa to extend patients’ lives.

The development of PCa is a combined result of genetic and epigenetic alterations that result in the formation of multifocal heterogeneous lesions (Datta et al., 2016; Arcila-Ruiz et al., 2017). Evidence shows that autophagy plays a key role in PCa development, and numerous studies focusing on potential anticancer treatments are ongoing (Naponelli et al., 2015; Amaravadi et al., 2016). Autophagy normally is maintained at low levels in cellular homeostasis, but is strongly induced by stressful conditions such as anticancer drug treatments. Notably, autophagy has recently emerged as a key regulator of multiple aspects of cancer (Shintani and Klionsky, 2004; Singh et al., 2017), and its role in both cancer suppression and progression is still not completely clarified. In PCa, whether autophagy contributes to or opposes cell death is context-specific (Bennett et al., 2013; Elio et al., 2013). The pro-autophagic beclin1 gene is allelically deleted in many PCas, suggesting that autophagy may represent a tumor suppression mechanism in the prostate. Additionally, studies performed in epithelial PCa cells showed that autophagy may provide a survival mechanism in the prostate (Naponelli et al., 2015). Together, these data paradoxically indicate that induction or inhibition of autophagy could have effects against PCa, depending on context.

Experimental and clinical data indicate that the mTOR pathway, and its upstream regulators in the PI3K/PTEN/Akt cascade, are altered in a wide variety of human malignancies (Ciuffreda et al., 2010). PI3K/Akt/mTOR complex-1 (mTORC1) and the androgen receptor are two major drivers of PCa, and the PI3K/Akt/mTOR signaling pathway plays a key role in anticancer drug-mediated regulation of autophagy in human PCa cells (Wang et al., 2016; Kim et al., 2017; Lin et al., 2017). Previous studies have also revealed that the degree of activation of Akt/mTOR signaling components is important, as too little or too much signaling has different consequences depending on the PCa state (Farrow et al., 2014). Therefore, targeting critical components in this signaling cascade to regulate autophagy in PCa cells has become an attractive new therapeutic strategy in the treatment of PCa.

Qianlie Xiaozheng decoction (QLXZD), prepared by Prof. Youfang Liu, is a TCM formula that has been used to effectively treat advanced PCa patients (Yin et al., 2015). QLXZD is composed of seven herbs as shown in **Table 1**. This formula has been used clinically for over 10 years as a means to effectively alleviate the symptoms of advanced PCa, and improve the patient’s lifespan and quality of life. Because QLXZD is widely used in clinical medicine, it is essential to elucidate its underlying mechanisms to understand its function as well as the basis of the PCa itself.

**Table 1:** Composition of QLXZD.

Species	Herbal name	Place of origin	Part of use	Use amount (g)
*Astragalus membranaceus* (Fisch.) Bge. var. mongholicus (Bge.) Hsiao	Astragali Radix Praeparata Cum Melle	Neimenggu	Root	15
*Polygonatum sibiricum* Red.	Polygonati Rhizoma	Hebei	Root and rhizoma	10
*Coix lacryma-jobi* L. var. mayuen (Roman.) Stapf	Coicis Semen	Hubei	Seed	30
*Curcuma phaeocaulis* Val.	Curcumae Rhizoma	Sichuan	Root and rhizoma	9
*Bolbostemma paniculatum* (Maxim.) Franquet	Bolbostemmatis Rhizoma	Henan	Tuber	9
*Polyporus umbellatus* (Pers.) Frie	Polyporus	Shanxi	Sclerotium	15
*Oldenlandia diffusa* (willd.) roxb	Oldenlandia diffusa (willd.) roxb	Fujian	Herba	20
**Total amount**				108

In this study, we examined the mechanism of QLXZD *in vitro* using the PC3 cell line. We also evaluated its safety and antitumor effect *in vivo* using healthy and PC3 tumor-bearing nude mice. The objectives of this research were to provide scientific evidence to support the use of QLXZD in the clinical treatment of PCa and to offer insight into its molecular mechanism.

## Materials and Methods

### Chemicals and Reagents

Seven herbs formed QLXZD were provided by Affiliated Hospital of Integrated Traditional Chinese and Western Medicine, Nanjing University of Chinese Medicine (Nanjing, Jiangsu, China). The seven crude drugs were morphologically authenticated according to Chinese Pharmacopoeia (2015 Edition). Reference substances including Calycosin-7-*O*-β-d-glucoside, rutin, calycosin, tubeimoside, formononetin and curdione, ergosterol were purchased from Must Biological Technology Co. Ltd. (Chengdu, China). The purity of each reference compound was over 98% by HPLC. HPLC-grade acetonitrile and methanol were purchased from ROE Scientific Inc. (United States). Deionized water was purified using a Milli-Q water purification system from Millipore (Bedford, MA, United States). The following antibodies were used: LC3B (Abcam, Cambridge, MA, United States); p62, mTOR, phospho-mTOR (Ser2448), p70S6K, phospho-p70S6K (T389), AKT, phospho-AKT (T308) (Cell Signaling Technology, Beverly, MA, United States); polyclonal rabbit anti-glyceraldehyde-3-phosphate dehydrogenase (GAPDH) (Bioworld, Technology, Inc., St. Louis Park, MN, United States). MTT (methyl thiazolyl tetrazolium), 3-MA, and HCQ were purchased from Sigma-Aldrich (St. Louis, MO, United States). FlowCellect^TM^ Autophagy LC3 Antibody-based Assay Kit was purchased from Millipore (Bedford, MA, United States). Other chemicals were provided by local commercial sources and were of analytical grade quality.

### QLXZD Preparation and Quality Control Analysis

To prepare QLXZD samples, 15 g of Astragali Radix Praeparata Cum Melle, 10 g of Polygonati Rhizoma, 30 g of Coicis Semen, 9 g of Curcumae Rhizoma, 9 g of Bolbostemmatis Rhizoma, 15 g of Polyporus, 20 g of Oldenlandia diffusa (willd.) roxb were soaked together in 10 volumes of distilled water for 30 min, then refluxed for 2 h (Cheng et al., 2017). The water extract was filtered, and the residue with eight volumes of water refluxed again for 2 h. The combined filtrates were desiccated to 1.08 g crude drug/mL, and stored at -80°C. Before quality control analysis, the extracts were diluted and filtered through a 0.22 μm filter membrane. Qualitative analysis was performed by Waters e2695 Alliance HPLC system (Waters Corp., Milford, MA, United States) with 2489 UV/Vis DAD detector. Chromatographic separation was carried out at 25°C on an Inertsil ODS-SP C18 column (250 mm × 4.6 mm, 5 μm) with 30 μL injection volume. The mobile phase consisted of linear gradients of 0.05% (v/v) phosphoric acid (A) and acetonitrile (B): 0–15 min, 2–5% B (v/v); 15–30 min, 5–10% B; 30–60 min, 10–40% B; 60–70 min, 40–75% B; 70–75 min, 75–100% B; 75–80 min, 100–100% B. The mobile phase flow rate was 1 mL/min and wavelengths were set at 254 and 210 nm. The concentrations of major constituents were determined by an external standard curve.

### Cell Lines and Cultures

PC3 was established from a grade 4 prostatic adenocarcinoma from a 62-year-old Caucasian male. DU 145 was isolated from a brain metastases lesion in a 69-year-old Caucasian male with PCa. PC3 and DU 145 cells were purchased from the Cell Bank of the Shanghai Institute of Biochemistry and Cell Biology and maintained in Ham’s F12 and MEM medium (Gibco, Life Technologies, Carlsbad, CA, United States) respectively, which supplemented with 10% fetal bovine serum (FBS) (Invitrogen, Carlsbad, CA, United States). All cells were cultured in a humidified atmosphere with a 5% CO_2_ incubator at 37°C.

### Cell Viability Assay

PC3 cells and DU145 cells were incubated in triplicate in a 96-well plate at a density of 1 × 10^4^ cells with 100 μL culture medium per well supplemented with increasing concentration of QLXZD (0–54 mg/mL) for 24 or 48 h. After which, cell viability was determined by the MTT dye uptake method as described earlier (Yang et al., 2016).

### Flow Cytometry Detection of LC3-II

Seed PC3 cells in six-well plates at a density of 5 × 10^5^ cells per well, in a CO_2_ incubator overnight and treated with QLXZD (0–30 mg/mL) in the presence or absence of 3-MA and H-CQ for 24 h. After which, autophagosome-associated LC3 assay by flow cytometry was performed as the protocol of FlowCellect^TM^ autophagy LC3 antibody-based Assay Kit (Millipore, Bedford, MA, United States).

### Transmission Electron Microscopy (TEM) Observation

Seed PC3 cells in six-well plates at a density of 5 × 10^5^ cells per well, in a CO_2_ incubator overnight and treated with QLXZD (0–30 mg/mL) for 24 h. The cells were collected and fixed in glutaraldehyde (2.5% in 0.1 mol/L cacodylate buffer, PH 7. 4) for 2 h at room temperature and postfixed in osmium tetroxide. After dehydration with a graded series of alcohol concentrations, the samples were rinsed in propylene oxide and impregnated with epoxy resins. The ultrathin sections were contrasted with uranyl acetate and lead citrate for electron microscopy. Electron micrographs were observed through a transmission electron microscope (H7650; Hitachi, Tokyo, Japan), according to the method as described earlier (Wang et al., 2013).

### Evaluation of Fluorescent LC3 Puncta

PC3 cells were cultured on coverslips for 24 h followed transfected with pmCherry-GFP-LC3 plasmid using Lipofectamine^TM^ 2000 (Invitrogen, Carlsbad, CA, United States) according to the manufacturer’s instruction for another 24 h before receiving QLXZD (0–30 mg/mL) treatments. After 24 h QLXZD treatment, the cells were washed with PBS, fixed with 4% paraformaldehyde, nuclei stained with hoechst and imaged with a confocal fluorescence microscopy (Olympus FV10i, Japan). The number of GFP and mRFP dots was determined as the method described earlier (Hariharan et al., 2011).

### Western Blot Analysis

Seed PC3 cells in six-well plates at a density of 5 × 10^5^ cells per well, in a CO_2_ incubator overnight and treated with QLXZD (0–30 mg/mL) for 24 h. The total protein were collected as previously described (Liu et al., 2017). The equalized amounts of proteins from each sample were subjected to sodium dodecyl sulfate polyacrylamide gel electrophoresis (SDS-PAGE) followed by transfer to polyvinylidene fluoride (PVDF) membranes. After blocking with 1% (w/v) albumin from bovine serum (BSA) for 2 h, the membranes were incubated overnight at 4°C with primary antibodies, followed by IRDye-conjugated secondary antibodies for 1 h at room temperature and scanned with an Odyssey infrared fluorescent scanner (LI-COR Biosciences). GAPDH was used as the endogenous control.

### Animals and Antitumor Activity *in Vivo*

Human prostatic cancer xenografts were established by injecting 5 × 10^6^ PC3 cells subcutaneously into 4-week-old BALB/c nude male mice. When the tumors reached a volume of 50–100 mm^3^, the mice were randomly assigned to model, positive control, and treated groups (low-dose group and high-dose group), and there was a control group that did not receive xenografts. The model group and the control group were given 0.2 mL of sterilized water twice 1 day via oral gavage, the positive control group was given 15 mg/kg CTX daily via oral gavage in a volume of 0.2 mL, the low-dose group was given 0.2 mL of a solution containing 108 mg of QLXZD twice per day via oral gavage (10.8 g/kg), and the high-dose group was given 0.2 mL of a solution containing 216 mg of QLXZD twice per day via oral gavage (21.6 g/kg). Body weight and tumor volume were measured individually every 2 days. Tumor volume was calculated using the formula: Volume = (width^2^ × length)/2 (Li et al., 2014). After 14 days, the mice were sacrificed and the organs and xenografts were removed for H&E staining and immunohistochemical staining, western blot assays, respectively. All animal experiments were conducted in accordance with the U.S. NIH Guidelines for the Care and Use of Laboratory Animals.

### Statistical Analysis

Statistical analysis was performed using SPSS SPSS 15.0 (SPSS, Inc., Chicago, IL, United States). Data are expressed as mean ± SD. Unpaired Student’s *t*-test were used to compare the means of two groups. Tests were two-sided and *P* < 0.05 was considered to indicate a statistically significant difference.

## Results

### Quality Control Analysis of QLXZD

**FIGURE 1:**
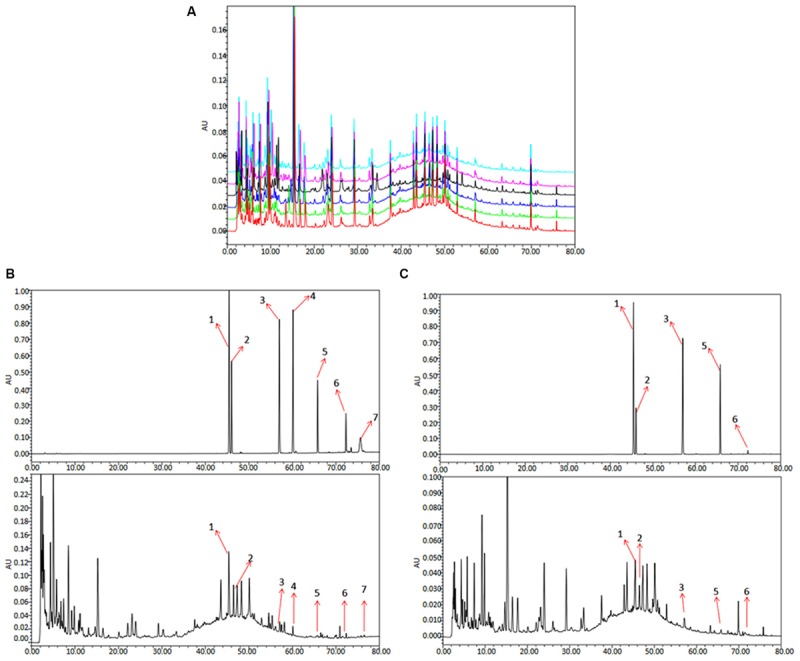
HPLC chromatogram of QLXZD. **(A)** Overlapping chromatograms of six batches of QLXZD. **(B)** Chromatogram at λ_max_ = 210 nm. The reference standards are shown on top, while QLXZD is shown below. **(C)** Chromatogram at λ_max_ = 254 nm. The reference standards are shown on top, while QLXZD is shown below.

To analyze and assess the quality of the QLXZD used in this study, chemical fingerprinting and quantitation of phytochemical markers were performed using chromatography. The species and concentrations of the seven herbs found in QLXZD are listed in **Table 1**. Overlapping chromatograms of the six batches of QLXZD used during this study are shown in **Figure 1A**. Importantly, the quality of the QLXZD used appeared to remain stable throughout the *in vitro* and *in vivo* experiments performed.

Chromatograms of the seven herbal constituents of QLXZD were analyzed quantitatively at 210 nm (**Figure 1B**) and 254 nm (**Figure 1C**) using an external standard curve, and the calculated concentrations (mg/L) of each compound are summarized in **Table 2**. The contents of calycosin-7-*O*-β-D-glucoside, tubeimoside, and rutin were greatest in QLXZD, and come, respectively, from Astragali Radix Praeparata Cum Melle, Bolbostemmatis Rhizoma, and Oldenlandia diffusa (willd.) roxb.

**Table 2:** Characterization of compounds in QLXZD.

Peak no.	Compound	Formula	Content (mg/L)	Source
1	Calycosin-7-*O*-β-D-glucoside	C_22_H_22_O_10_	20.493	Astragali Radix Praeparata Cum Melle
2	Rutin	C_27_H_30_O_16_	7.012	Oldenlandia diffusa (willd.) roxb
3	Calycosin	C_16_H_12_O_5_	1.793	Astragali Radix Praeparata Cum Melle
4	Tubeimoside I	C_63_H_98_O_29_	15.6	Bolbostemmatis Rhizoma
5	Formononetin	C_16_H_12_O_4_	0.286	Astragali Radix Praeparata Cum Melle
6	Curdione	C_15_H_24_O_2_	2.259	Curcumae Rhizoma
7	Ergosterol	C_28_H_44_O	0.193	Polyporus

### Suppression of Cell Viability by QLXZD in Human PCa Cell Lines

**FIGURE 2:**
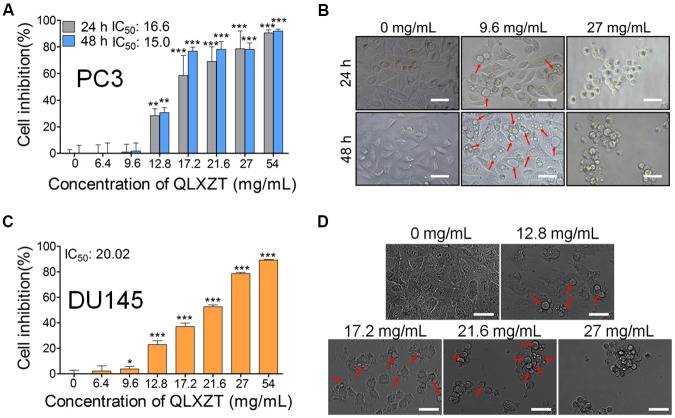
Viability of human PCa cell lines treated with QLXZD. **(A)** PC3 cells were treated with the specified concentrations of QLXZD for 24 and 48 h, and then subjected to MTT assay. Results are expressed as means ± SD (*n* = 3). Statistical differences between groups were analyzed by Student’s *t*-test, ^∗∗^*P* < 0.01, ^∗∗∗^*P* < 0.001 compared to the control (0 mg/mL of QLXZD). **(B)** Representative images of PC3 cell morphology after treatment with the specified concentrations of QLXZD for 24 and 48 h. Scale bar = 50 μm. **(C)** DU145 cells were treated with the specified concentrations of QLXZD for 24 h and then subjected to the MTT assay. The results are expressed as means ± SD (*n* = 3). Statistical differences between groups were analyzed by Student’s *t*-test, ^∗^*P* < 0.05, ^∗∗∗^*P* < 0.001 compared to the control (0 mg/mL of QLXZD). **(D)** Representative images of DU145 cell morphology after treatment with the specified concentrations of QLXZD for 24 h. Scale bars = 50 μm.

To evaluate the cytotoxicity of QLXZD, PC3 cells were treated with various concentrations of QLXZD for 24 and 48 h. QLXZD inhibited PC3 cell viability significantly in a time- and concentration-dependent manner (**Figures 2A,B**). The IC_50_ values of QLXZD for PC3 cells treated for 24 and 48 h were 16.6 and 15.0 mg/mL, respectively. PC3 cells treated with 9.6 mg/mL showed no morphological changes, but suspected autophagic vacuoles (red arrows) were observed, especially in cells treated for 48 h (**Figure 2B**). As shown in Supplementary Figure S1, treatment with autophagy inhibitors compromised QLXZD-induced cytotoxicity in PC3 cells. The cells appeared obviously lower in density and shrunken following treatment with 27 mg/mL QLXZD.

QLXZD inhibited DU145 cell viability significantly at 24 h in a concentration-dependent manner (**Figures 2C,D**). The IC_50_ value of QLXZD for DU145 cells treated for 24 h was 20.0 mg/mL. DU145 cells treated with 12.8 mg/mL showed no morphological changes, but suspected autophagic vacuoles (red arrows) were observed, especially in cells treated with 17.2 mg/mL (**Figure 2D**). Cells treated with 21.6 and 27 mg/mL appeared obviously lower in density and shrunken. Based on these findings, a 24 h exposure time and PC3 cells were used for all subsequent experiments.

### Autophagy in PC3 Cells Induced by QLXZD

**FIGURE 3:**
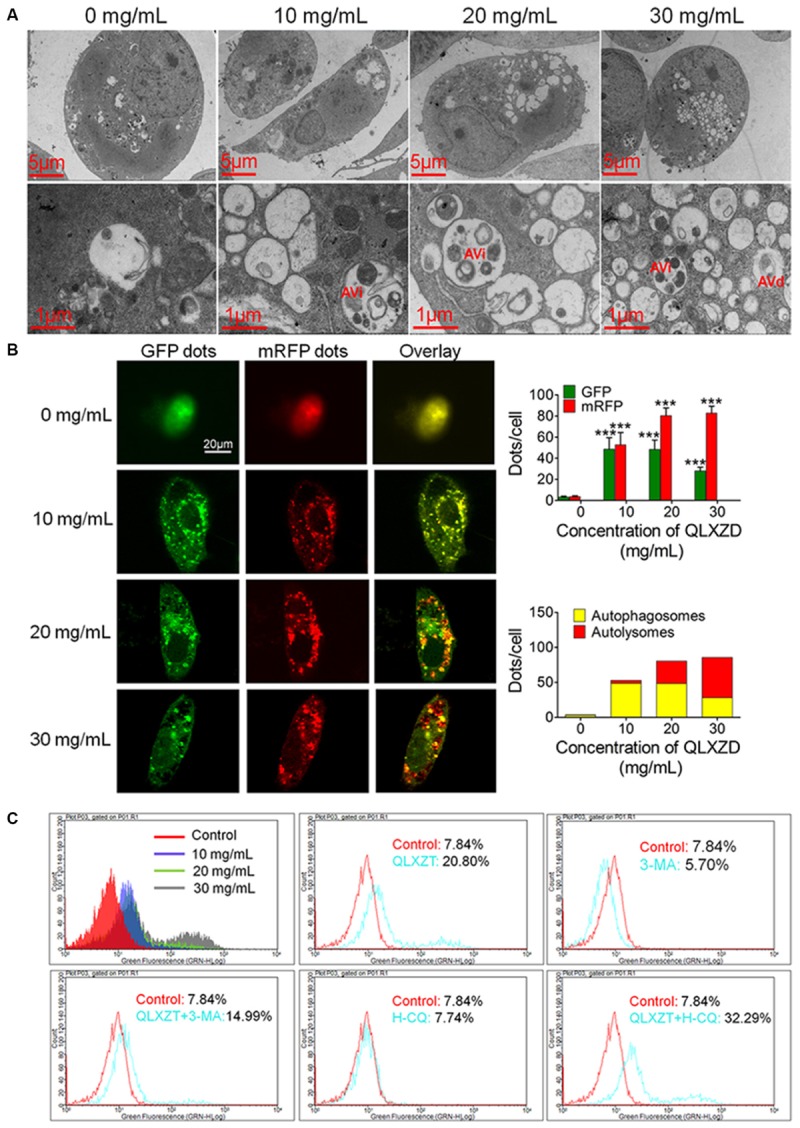
Autophagy in PC3 cells induced by QLXZD. **(A)** Representative TEM images. Autophagic vacuoles were monitored in PC3 cells and increased with the increasing of QLXZD concentration (scale bar = 5 μm). Initial autophagic vacuoles (AVis) and degradative autophagic vacuoles (AVds) are also highlighted (scale bar = 1 μm). **(B)** Representative confocal microscopy images of PC3 cells transfected with fluorescent Cherry-GFP-LC3 plasmid after QLXZD treatment. QLXZD treatment increased both numbers of GFP and mRFP dots per cell. ^∗∗∗^*P* < 0.001. **(C)** FACS was used to detect GFP-LC3 expression in QLXZD-treated PC3 cells as well as cells treated with 3-MA and QLXZD (which induced a left-ward shift of the histogram) or HCQ and QLXZD (which induced a right-ward shift of the histogram).

Transmission electron microscopy can be used to identify autophagic structures morphologically at a resolution in the nm range in their natural environment and cellular position (Klionsky et al., 2016). TEM analysis of QLXZD-treated PC3 cells showed the formation of distinct autophagic vacuoles that appeared to be dependent on the QLXZD concentration (**Figure 3A**, scale bar = 5 μm). Furthermore, AVi could be differentiated from AVd in QLXZD-treated cells using this analysis (**Figure 3A**, scale bar = 1 μm).

Because autophagic flux is a dynamic process, it is imperative to differentiate between increased autophagosome formation and decreased autolysosome clearance to determine the true level of autophagy (Hariharan et al., 2011). pmCherry-GFP-LC3 fluorescence analysis is a well-characterized method used to monitor autophagic flux (Kimura et al., 2007; Jacquin et al., 2017). In this analysis, GFP fluorescence, but not mRFP fluorescence, is quenched at the acidic pH of autolysosomes, while both GFP and mRFP continue to fluoresce in autophagosomes. Therefore, an increase in autophagic flux is indicated by a higher ratio of red (mRFP alone: autolysosomes) to yellow (mRFP and GFP merged: autophagosomes) punctate staining. As shown in **Figure 3B**, QLXZD treatment increased the total number of both autophagosomes and autolysosomes per cell. Furthermore, treating the cells with 30 mg/mL QLXZD significantly increased the ratio of autolysosomes to autophagosomes (more free red than yellow puncta were seen), suggesting that QLXZD increased autophagic flux.

Flow cytometry was performed to evaluate the autophagy-associated expression of GFP-labeled LC3-II. These data indicated that the percentage of QLXZD-treated cells expressing GFP-LC3-II increased significantly in a concentration-dependent manner compared with that in the control group, as illustrated by the rightward shift of the histogram (**Figure 3C**).

To confirm that the observed increase in LC3-II-expressing PC3 cells was indeed related to autophagy, the cells were treated with two known autophagic inhibitors: 3-MA and HCQ. Treating PC3 cells with HCQ (30 μM) and QLXZD (20 mg/mL) simultaneously for 24 h resulted in a rightward shift of the histogram compared with PC3 cells treated with QLXZD alone. This indicated inhibition of QLXZD-induced autophagy due to its inhibition of autolysosome degradation. Alternatively, pre-treating cells with 3-MA (5 mM) for 2 h before the addition of QLXZD (20 mg/mL) for 24 h resulted in a leftward shift of the histogram compared with PC3 cells treated with QLXZD alone due to the inhibition of autophagosome formation.

### Inhibition of the PI3K/Akt/mTOR Signaling Pathway in PC3 Cells

**FIGURE 4:**
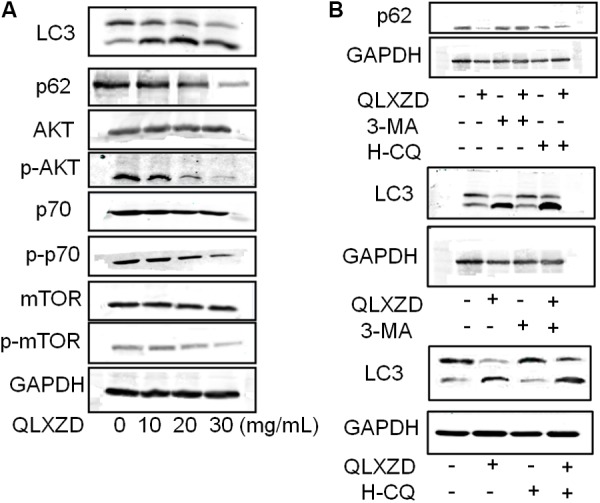
Inhibition of the PI3K/Akt/mTOR signaling pathway in PC3 cells. **(A)** After treatment with QLXZD, the expression of LC3-I/II and p62 were detected by western blotting, a dose-dependent decrease of phosphorylation of proteins involved in the Akt/mTOR signaling pathway were observed. **(B)** After treatment with QLXZD, 3-MA and HCQ, the expression of LC3-I/II and p62 were detected by western blotting.

The expression of two key markers of autophagy, LC3-II and p62, was examined by western blotting (Jiang and Mizushima, 2015; Feng et al., 2017). QLXZD treatment appeared to drive the conversion of LC3 from the cytoplasmic form (LC3-I) into the autophagosomic form (LC3-II) while also decreasing the expression of p62 in PC3 cells in a concentration-dependent manner (**Figure 4A**). PC3 cells treated with 20 mg/mL QLXZD were also treated with HCQ or 3-MA as described above. HCQ treatment inhibited the degradation of LC3-II and p62 in autolysosomes, while 3-MA treatment significantly inhibited the degradation of p62 and conversion of LC3-II (**Figure 4B**). These data further confirmed that QLXZD induced autophagic cell death in PC3 cells.

To investigate whether QLXZD inhibited the PI3K/Akt/mTOR signaling pathway, we used western blotting to assess the expression of various proteins involved in this signaling cascade (Akt, p-Akt, mTOR, p-mTOR, p70S6K, and p-p70S6K) in PC3 cells treated with QLXZD. As shown in **Figure 4A**, QLXZD concentration-dependently inhibited the phosphorylation of Akt (T308), mTOR (Ser2448), and p70S6K (T389) in PC3 cells, while the total expression of these proteins was not altered by QLXZD treatment. Inhibiting the phosphorylation-dependent activation of these proteins likely plays a significant role in the observed QLXZD-induced autophagy in PC3 cells.

### Inhibitory Effect of QLXZD on PC3 Xenograft Growth *in Vivo*

**FIGURE 5:**
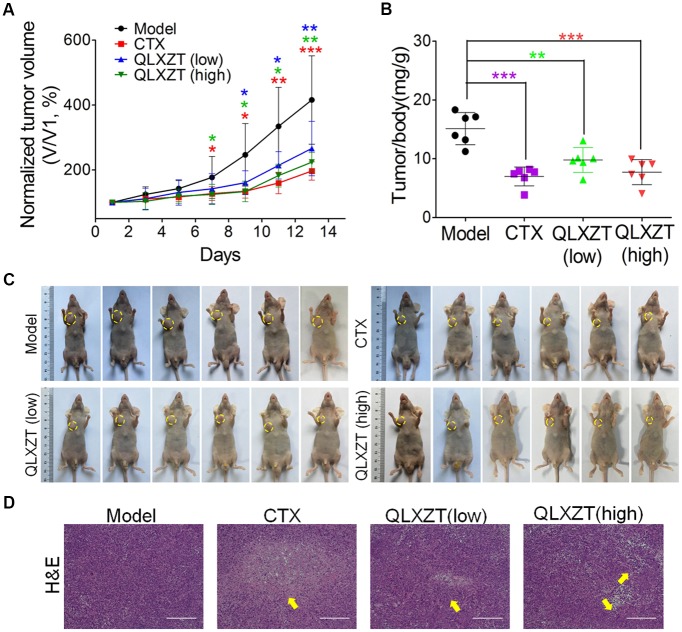
Antitumor activity *in vivo* of QLXZD against PC3 xenografts. The tumor growth curve **(A)** and the ratio curve of tumor to body weight of nude mice **(B)**. Statistical differences between treating groups and model group were analyzed by Student’s *t*-test, ^∗^*P* < 0.05, ^∗∗^*P* < 0.01, ^∗∗∗^*P* < 0.001, the values are expressed as the mean ± SD (*n* = 6). **(C)** The inhibition in the size of the xenografted prostate tumors is shown, yellow circle indicate the tumors on mice. **(D)** Representative images of H&E stained xenograft tumor tissue indicate that high doses of QLXZD caused obvious tumor inhibition (yellow arrow). Scale bar = 200 μm.

Mice bearing PC3 xenografts were treated with QLXZD, and their body weights and tumor volumes were measured individually every 2 days. After 14 days of treatment, the mice were sacrificed, and their organs and xenografts were moved and weighed. QLXZD treatment caused a marked inhibition of tumor growth compared with vehicle-treated mice (**Figure 5A**). Tumors grew rapidly in mice treated with sterilized water. By day 14, the average tumor volume in the model group increased by approximately fourfold, whereas the tumor volume in QLZXD-treated mice increased only twofold. This was approximately 46% less than the control model group, and similar to the CTX-treated group. The inhibition of tumor growth in mice treated with the high concentration of QLXZD was slightly greater than that in the group treated with the low concentration. Differences in tumor volumes of the groups treated with QLXZD (*P* < 0.01) and CTX (*P* < 0.001), when compared with that of the model group, were both statistically significant. Changes in the tumor to body weight ratio (**Figure 5B**) were consistent with the changes in tumor volume. Differences in the tumor to body weight ratio in the CTX-treated group (*P* < 0.001), high-concentration QLXZD-treated group (*P* < 0.001), and low-concentration QLXZD-treated group (*P* < 0.01), when compared with the model group, were statistically significant.

Photographs of complete tumors in mice are shown in **Figure 5C** (marked with yellow circles). Xenograft tumor tissues were also examined by hematoxylin and eosin staining (**Figure 5D**). The pathology examination clearly demonstrated tumor necrosis in the CTX- and QLXZD-treated groups (yellow arrows), but not in the model group. The high-concentration QLXZD-treated group showed much larger necrotic regions than the low-concentration QLXZD-treated group; the CTX group had the largest necrotic area (**Figure 5D**).

### Autophagy Induced by QLXZD in PC3 Xenografts *in Vivo*

**FIGURE 6:**
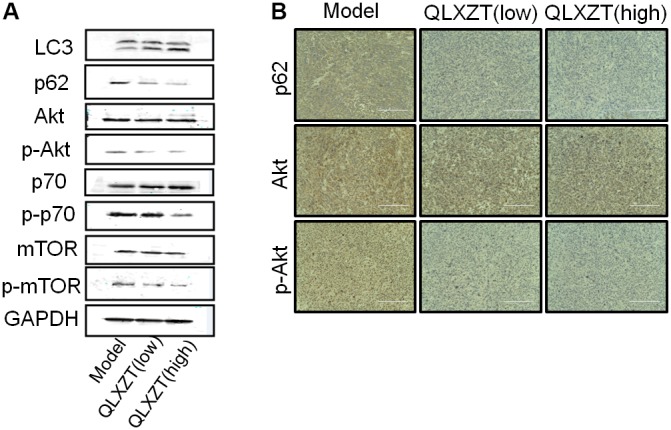
Autophagy induced by QLXZD via inhibiting PI3K/Akt/mTOR signaling in PC3 xenografts. **(A)** Isolated xenograft tumor tissue was subjected to western blotting using anti-LC3 and anti-p62 antibodies, and the expression of Akt, p-Akt, mTOR, p-mTOR, p70S6K, and p-p70S6K was also analyzed with western blot. **(B)** Representative immunohistochemistry images of the xenograft tumor tissue showing cells that were positive (brown staining) and negative (blue staining) for p62, AKT, p-AKT. Scale bar = 200 μm.

Isolated xenograft tumor tissue was subjected to western blotting (**Figure 6A**) and immunohistochemical analysis (**Figure 6B**). QLXZD was again observed to increase the conversion of LC3-I to LC3-II and decrease the expression of p62 in a dose-dependent manner. Furthermore, the expression of PI3K/Akt/mTOR pathway-related proteins was also altered in xenograft tumor tissue isolated from QLXZD-treated mice, with the phosphorylation of Akt (T308), mTOR (Ser2448), and p70S6K (T389) being inhibited in the QLXZD-treated group. Similar to our *in vitro* experiments, total levels of these proteins were not altered regardless of treatment, confirming the role of PI3K/Akt/mTOR pathway inhibition during QLXZD-induced autophagy.

### No *in Vivo* Aberrant Side-Effects Were Caused by QLXZD Treatment

**FIGURE 7:**
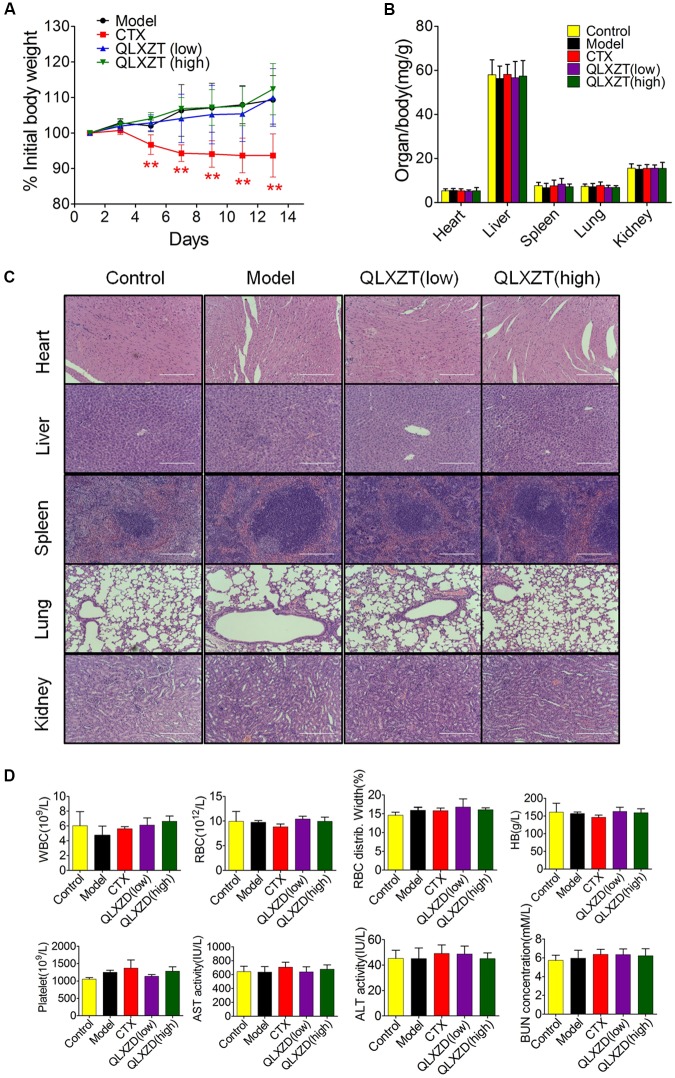
*In vivo* safety evaluation of QLXZD. **(A)** The body weight curve of all test groups. Statistical differences between treating groups and model group were analyzed by Student’s *t*-test, ^∗∗^*P* < 0.01, the values are expressed as the mean ± SD (*n* = 6). **(B)** The ratio of major organs to body weight in all groups. Statistical differences between test groups and control group were analyzed by Student’s *t*-test, the values are expressed as the mean ± SD (*n* = 6). **(C)** Representative images of H&E stained sections of major organs. Scale bar = 200 μm. **(D)** Hematological parameters and serum levels of AST, ALT, and BUN. Data are shown as the means ± SD (*n* = 3).

Mice were weighed individually every 2 days over the 14-day course of treatment. The increase in body weight observed in QLXZD-treated mice was consistent with the control group, while CTX-treated mice showed a significant decrease in body weight (**Figure 7A**). Major organs were harvested immediately after the mice were sacrificed. The organ to body weight ratios showed no significant differences between treatment groups (**Figure 7B**). No obvious histological differences were found (**Figure 7C**).

Blood samples were collected before mice were sacrificed and used for routine blood tests within 15 min. We found no physiologically significant differences in any of the important hematology markers or in the blood serum concentrations of aspartate transaminase, alanine transaminase, or blood urea nitrogen in the QLXZD-treated mice (**Figure 7D**). Taken together, these data suggest these doses of QLXZD were non-toxic *in vivo*.

## Discussion

According to the serum androgen level of PCa patients, PCa is divided into HDPC and HIPC in WHO classification (Newling et al., 1997). The primary PCa is almost the typical HDPC, androgen is the key growth factor of which, and androgen deprivation therapy shows obvious effects at the beginning of treatment. As the course of the PCa progresses after 14–30 months, most patients develop HIPC and eventually die from it, which is the final form and inevitable result of the development of PCa, and there is currently no effective treatment or cure (Thy et al., 2015; Raymond et al., 2017). Among the various PCa cells, androgen-refractory PC3 and DU 145 cells are widely used in HIPC research. PC3 cell line was isolated from a grade 4 prostatic adenocarcinoma from a 62-year-old Caucasian male. DU 145 cell line was isolated from a brain metastases lesion in a 69-year-old Caucasian male with PCa and 3-year history of lymphocytic leukemia. Studies have shown that the two well-characterized androgen independent cell lines, which lack androgen receptors, exhibit differences in their chemotherapeutic response (Jayakumar et al., 2014). Some sublines of PC3 cell line are highly metastatic when grafted to nude mice, while DU 145 is moderately metastatic in comparison to that of PC3 cells (Janssen et al., 2015).

Traditional Chinese medicine has been used extensively in the treatment of patients with metastatic PCa develop HIPC; it has multiple targets and action mechanisms, and varying degrees of efficacy depending on the synergistic actions of its complex chemical components (Liu et al., 2015). QLXZD prepared by Prof. Youfang Liu is an effective TCM prescription and has been used clinically as a treatment for advanced PCa for more than 10 years. Modern medical research suggests that autophagic modulation by current PCa treatments is a promising therapeutic option (Farrow et al., 2014). QLXZD may be particularly useful for patients with HIPC because it ameliorates urinary urgency, dysuria, and weakness, and decreases the doubling time of prostate-specific antigen levels, thereby benefitting patients. In this study, we performed a quality control analysis of this TCM and evaluated its effects *in vitro* and *in vivo* to better understand its underlying mechanism. We demonstrated, for the first time, both the mechanism and efficacy of QLXZD as a treatment option for PCa.

It is important to note that autophagy plays a paradoxical role during PCa progression; it can be neutral, tumor-suppressive, or tumor-promoting in different contexts. In this study, we demonstrate that QLXZD indeed has antitumor capabilities and induces autophagy in PC3 cells *in vitro* and in PC3 xenografts *in vivo* with no overt toxicity. Autophagic vacuole formation and autophagic flux were monitored in QLXZD-treated PC3 cells using TEM, and by assessing the protein levels of LC3-I/II and p62. The responses appeared to be largely concentration-dependent and were inhibited by 3-MA and HCQ, and no significant increase of apoptosis was involved in QLXZD-induced effects (Supplementary Figure S2). Indicating that induction of autophagy was the main pathway regulated by QLXZD. These results are consistent with the literature regarding PCa treatment via autophagy induction, but opposite to the autophagy blockade induced by capsaicin (a natural ingredient in peppers) in PCa cells (Jiang et al., 2013; Kumar et al., 2014). These results confirm that autophagy plays a paradoxical role during PCa progression. Thus, the final effect of autophagy in cancer cells is highly variable depending on the integration of extracellular conditions and complex signaling pathways.

To further elucidate the mechanism by which QLXZD regulated autophagy, we performed various molecular studies focusing on the mTOR pathway. Numerous reports have demonstrated that mTOR kinase suppresses autophagy and apoptosis (Wang et al., 2015) via interference with Atg protein-related formation of autophagosomes (Levine and Yuan, 2005). The PI3K/Akt/mTOR signaling pathways are key axes involved in anticancer drug-induced autophagy, including PCa treatment (Kim et al., 2017; Lin et al., 2017; Yu et al., 2017). This largely explains the effectiveness of mTOR inhibitors, such as rapamycin, as anticancer agents. However, rapamycin only inhibits mTORC1, whereas its upstream regulators are still activated (Yori et al., 2014; Fraga et al., 2017). This limits its antineoplastic effects. Our data indicated that QLXZD not only inhibited the activation of mTORC1 but also blocked the phosphorylation of its upstream regulator, namely Akt, which prevented compensatory feedback from its downstream regulator. Therefore, it appears that QLXZD may be a stronger antitumor drug than other conventional therapeutics. Although additional work is necessary to fully characterize the clinical efficacy of QLXZD and other TCMs, these findings highlight a future for this QLXZD formula in the treatment of androgen-independent PCa.

## Conclusion

**FIGURE 8:**
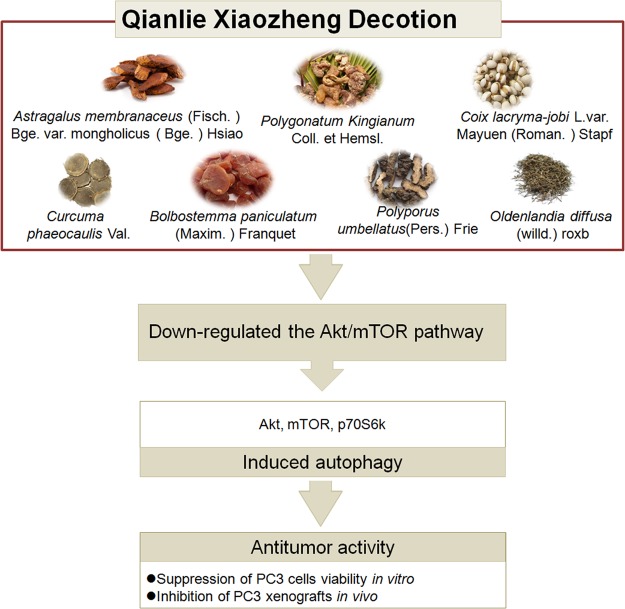
The ingredients, antitumor activity against prostate cancer and possible mechanisms of action of QLXZD. The decoction is made from seven different herbs. Its antitumor activity against prostate cancer *in vitro* and *in vivo* is proposed to be due to its autophagy induction via down-regulating the AKT/mTOR pathway.

This study is the first to demonstrate the mechanistic pathways utilized during QLXZD-mediated antitumor activity both *in vitro* (PC3 cells) and in xenografts (**Figure 8**). These effects are mediated through induction of excessive autophagy at high concentrations of QLXZD, leading to accelerated cell death. QLXZD functions by inhibiting the Akt/mTOR signaling pathway. These findings provide experimental evidence for the clinical use of QLXZD and a foundation for further scientific study of TCMs.

## Author Contributions

YX obtained funding, design, acquisition of data, analysis of data, and writing of the manuscript. XC design, technical support, proof-reading, and revision of the manuscript. BZ technical support and revision of the manuscript. RF, YJ, and GC technical support, analysis and interpretation of data. ZL obtained funding, conception and design, study supervision, writing and revision of the manuscript. All authors read and approved the final manuscript.

## Conflict of Interest Statement

The authors declare that the research was conducted in the absence of any commercial or financial relationships that could be construed as a potential conflict of interest.

## Abbreviations

3-MA: 3-methyladenine
AVd: degradative autophagic vacuole
AVi: initial autophagic vacuoles
CTX: cyclophosphamide
GFP: green fluorescent protein
HCQ: hydroxychloroquine
HDPC: hormone dependent PCa
HIPC: hormone-independent PCa
mRFP: monomeric red fluorescent protein
mTORC1: mechanistic target of rapamycin complex-1
PCa: prostate cancer
PI3K: phosphoinositide 3-kinase
QLXZD: Qianlie Xiaozheng decoction
TCM: traditional Chinese medicine
TEM: transmission electron microscopy.
