# Supplementary material for: Qianlie Xiaozheng Decoction Induces Autophagy in Human Prostate Cancer Cells via Inhibition of the Akt/mTOR Pathway
Source: Front Pharmacol. 2018 Apr 4;9:234. doi: 10.3389/fphar.2018.00234 (PMC5893804; doi:10.3389/fphar.2018.00234)
Supplement: Supplementary file 1 [file Presentation_1.PDF]

Qianlie Xiaozheng decoction induces autophagy in human prostate cancer cells via inhibition of the Akt/mTOR pathway

Supplementary Materials

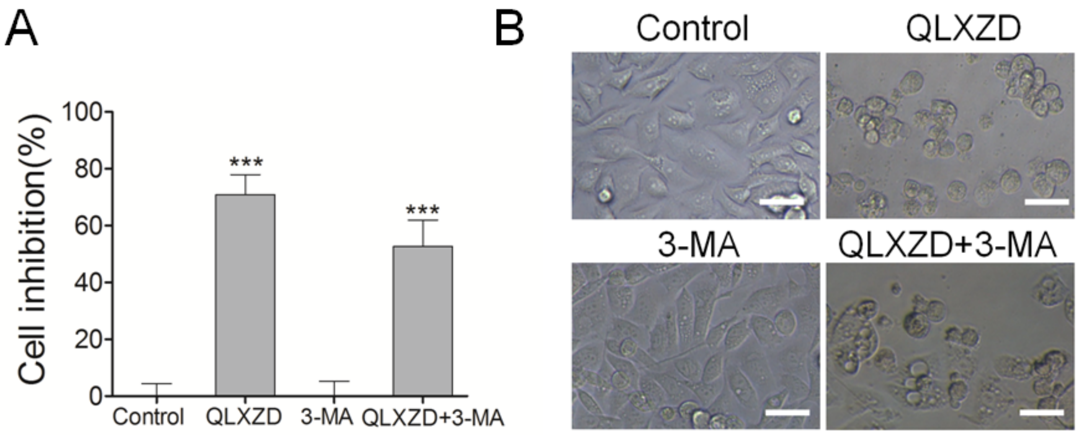

Supplementary Figure S1: Viability of PC3 cells. (**A**) PC3 cells were treated with QLXZD (20 mg/mL) and 3-MA (5 mM), and then subjected to MTT assay. Results are expressed as means ± SD (n = 3). Statistical differences between groups were analyzed by Student's t-test. $^{***}P < 0.001$ compared to the control (0 mg/mL QLXZD). (**B**) Representative images of PC3 cell morphology after treatment with QLXZD and 3-MA. Scale bars = 50 µm.

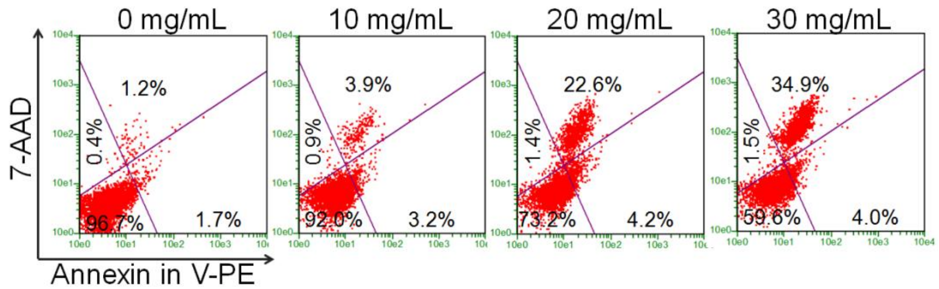

Supplementary Figure S2: Apoptosis in PC3 cells induced by QLXZD. FACS was used to detect apoptosis in QLXZD-treated PC3 cells after stained with Guava Nexin Reagent. The results show no significant increase in the percentage of early apoptotic cells (lower right). Dead cells (upper right) significantly increase in a concentration-dependent manner compared with the control group.
